# IMPORTANCE OF DIAGNOSTIC IMAGING FOR INTERPRETATION OF SELF-REPORTED SURVEYS OF SHOULDER FUNCTION

**DOI:** 10.1093/geroni/igz038.3268

**Published:** 2019-11-08

**Authors:** Derik L Davis, Ranyah Almardawi, Brock Beamer, Rao Gullapalli, Alice Ryan, Michael Terrin

**Affiliations:** 1 University of Maryland School of Maryland, Baltimore, Maryland, United States; 2 Baltimore VA Medical Center GRECC, Baltimore, Maryland, United States; 3 University of Maryland Baltimore School of Medicine, Baltimore, Maryland, United States

## Abstract

Rotator cuff (RC) tear is highly prevalent in older adults. The American Shoulder and Elbow Surgeon (ASES) survey, which quantifies subjective self-reported shoulder function, was originally validated in adults <60 years, and more recently is suggested to be valid in adults ≥60 years. We tested the hypothesis that ASES score (1) discriminates between adults 60—85 years with and without RC tear and (2) correlates with self-reported health quality and objective shoulder measures. Cross-sectional study: forty-two community-dwelling-older-adult volunteers (mean age, 69.4 ± 5.7 years; range, 61-84 years; male, 45%) with no history of RC surgery completed shoulder magnetic resonance imaging (MRI), shoulder forward flexion (FF) and abduction (ABD) range-of-motion (ROM) testing; and ASES, shoulder-pain and SF-36 (pain/physical function) surveys. Four groups (group-1:-pain,-tear,n=14; group-2:+pain,-tear,n=4; group-3:-pain,+tear,n=12; group-4:+pain,+tear,n=12) were compared using one-way ANOVA with ad hoc pairwise comparisons and Spearman Rank Order Correlation (rho). Age, Charlson co-morbidity index, and SF-36 pain/physical function were not appreciably different among all groups. ASES score (p<0.001), FF-ROM (p=0.032) and ABD-ROM (p=0.018) comparing all groups. ASES score: group-1 versus group-4 (p<0.001); but no difference between group-1 versus group-3 (p=0.999), and group-2 had lowest ASES score. ASES score correlated with SF-36 physical function (rho=0.47,p=0.002), SF-36 pain (rho=0.34,p=0.028), FF-ROM (rho=0.63,p<0.001), and ABD-ROM (rho=0.66,p<0.001). Our results suggest that additional research on ASES score in older adults is needed. Although valid, interpretation of ASES score in older adults should be approached cautiously in studies without shoulder diagnostic imaging tests, since painless RC tears and painful shoulders without RC tear are not rare.

